# Special Issue “Molecular Research on Embryo Developmental Potential”

**DOI:** 10.3390/ijms262411794

**Published:** 2025-12-06

**Authors:** Jan Tesarik

**Affiliations:** Molecular Assisted Reproduction and Genetics (MARGen) Clinic, 18002 Granada, Spain; jantesarik13@gmail.com; Tel.: +34-606376992

Along with the capacity of the uterus to promote implantation of the recently formed embryo (uterine receptivity), embryo developmental potential is another important factor for establishing normal pregnancy in both natural reproduction and assisted reproduction treatment (ART) attempts. Since most of the existing methods of embryo quality evaluation are either too invasive (decreasing the developmental potential even in normal embryos) or not reliable enough, the need for non-invasive methods using molecular biomarkers is evident. This Special Issue, containing 13 contributions, aims to collect relevant information on how to evaluate embryo developmental potential by minimally invasive means, based on experiments in humans and experimental models. Thirteen papers have been published, dealing with different species, from plants to humans.

To begin with, the original research article by Nowak et al. (Contribution 1) reports data obtained in a plant model (*Arabidopsis*) and shows an important role of micro-RNAs (miR156-SPL and miR169-NF-YA) in the regulation of embryogenesis. The other articles in this Special Issue are based on animal and human experiments.

Yusuf et al. (Contribution 2) explain how supraphysiological levels of testosterone lead to a significant reduction in estrogen and progesterone in rats, thus interrupting the short implantation window. These findings further reinforce the impact of androgens in endometrial receptivity and contribute to further developing androgen-targeted therapeutic approaches.

Fakhar-I-Adil et al. (Contribution 3) report that biphasic capacitation–in vitro maturation can improve equine oocyte quality and subsequent embryo development without inducing genetic aberrations. They claim that the equine serves as an excellent model for human reproduction. The molecular trends identified in this study could provide valuable insights for advancing human artificial reproductive technologies.

Shimura et al. (Contribution 4) address the question of whether the morphokinetics of second polar body formation in human zygotes can predict embryonic developmental potential via time-lapse observations.. The authors conclude that good-quality and euploid embryos usually have an unfragmented second polar body, as opposed to fragmenting and ruffling ones, thus adding a new predictor of embryonic developmental potential to be used in artificial intelligence-powered embryo evaluation systems.

Han et al. (Contribution 5) used nanopore-based sequencing of the full transcriptome of male and female cleavage-stage embryos of the Chinese Mitten Crab (*Eriocheir sinensis*) to analyze sex development in this species. The data obtained suggest that differential expression of ribosomal protein genes between genders, intrinsically linked to energy metabolism, may exert regulatory effects on gene expression and thereby regulate the difference in development between male and female embryos.

The experimental study by Costa et al. (Contribution 6), performed with bovine embryos, addressed the effects of C-type natriuretic peptide (CNP) supplementation at different stages of in vitro development. The presence of CNP receptors was detected from the germinal-vesicle-stage oocytes through blastocyst-stage embryos. When added to culture media throughout these stages, CNP improved blastocyst formation as compared to the control. These findings open novel perspectives for mammalian oocyte and embryo culture media improvement.

Cheng et al. (Contribution 7) focus on the specific expression profile and function of circular RNA (circRNA) derived from healthy and atretic follicles in buffalo and analyzed by RNA sequencing. The authors reveal the mechanism of differential expression of circRNA as both a regulator and marker of follicular atresia. These results provide new insights into the biological mechanisms underlying ovarian follicular atresia.

Li and Sun (Contribution 8) carried out an interesting investigation into the molecular mechanisms underlying anterior-to-dorsal eye rotation in the celestial-eye goldfish (*Carassius auratus*), a species subjected to millennia of artificial selection and breeding to obtain numerous ornamental eye varieties. In addition to previous studies focused on anatomical modifications (still poorly understood), this study employed high-throughput transcriptome and proteome sequencing of young sibling goldfish which displayed either anterior or upward eye rotations, using quantitative PCR for transcriptomic data and parallel reaction monitoring for proteomic analysis. These data strongly suggest the potential use of celestial-eye goldfish as a model organism for studying human eye-related disorders.

Li et al. (Contribution 9) explored conditions for the scaled application of laparoscopic ovum pick-up (LOPU) in sheep. They also performed transcriptomic analysis of follicular contents and metabolomic profiling of follicular fluid samples. Moreover, they evaluated the impact of the use of controlled internal drug release (CIDR), FSH source and dose, and recovery intervals. The best results were obtained in the CIDR group and with a 30-day recovery interval, whereas no significant differences were observed between FSH sources and hormone doses. The multi-omics analysis revealed that differentially expressed genes and metabolites were mainly those involved in steroidogenesis, and amino acid and fatty acid metabolism.

Yu et al. (Contribution 10) evaluated the presence of lysophosphatidic acid (LPA), a small bioactive phospholipid involved in early embryonic development, in bovine oviductal fluid and its receptors in bovine embryos. The findings were related to the degree of apoptosis in embryonic cells. LPA, assessed by enzyme-linked immunosorbent assay (ELISA), was found to be present in oviductal fluid. Additionally, an increase in total cell number and a decrease in apoptosis level, evaluated by terminal deoxynucleotidyl transferase nick end labeling (TUNEL) and quantitative reverse transcription polymerase chain reaction (qRT-PCR), were observed in day-7 blastocysts after LPA treatment. Out of eight LPA receptors detected in the blastocysts, only one (LPAR2) appeared to be involved in this effect. These data highlight the role of LPA in embryo development and point to possible signaling mediators.

The review article by Tesarik (Contribution 11) summarizes the current development of noninvasive biomarkers of human embryo developmental potential which are progressively replacing the traditional invasive ones, more harmful for embryonic viability and often less reliable. Some of these biomarkers are based on the direct assessment of embryo morphology across successive developmental stages, while others use spent media from embryo culture as the source of information. As compared with earlier methods for embryo quality evaluation, they use advanced high-output techniques, namely time-lapse recording (for direct embryo evaluation), next-generation sequencing, quantitative real-time polymerase chain reaction, high-performance liquid chromatography, nanoparticle tracking analysis, flow cytometry, and mass, Raman, near-infrared and nuclear magnetic resonance spectroscopies (for “omics” analyses of spent media). The current status, strengths and limitations of each technique are summarized.

The experimental study by Kontsevaya et al. (Contribution 12) evaluated the effects of the exposure of mouse embryos to hypothermia (35 °C) during zygotic genome activation (ZGA) on the formation of stress granules, preimplantation mortality and long-term phenotypic outcomes (developmental instability, behavioral deviation, hippocampal volume, and metabolomics profile of adult offspring). The short-term effects included an increased number and inter-blastomere variability of stress granules, extended duration of the second cleavage division, and increased embryonic mortality during the second and third cleavage stages. On the other hand, developmental instability of offspring resulting from the embryos exposed to hypothermic stress during ZGA was reduced, suggesting that embryos that have survived hypothermic exposure have a greater capacity to resist the adverse effects associated with the embryo transfer procedure. In addition, offspring resulting from embryos that had endured hypothermia during ZGA showed signs of a reduced risk of pathologies related to cognitive functions.

In their research article, Tsopp et al. (Contribution 13) used bovine embryos to test the hypothesis that metabolomic analysis of spent culture media could distinguish between high-quality early blastocysts and those with the same morphological appearance but undergoing developmental arrest at later stages. They used liquid chromatography–mass spectrometry to assess 189 metabolites in blastocysts that subsequently underwent successful hatching (viable blastocysts) and in those that failed to do so (non-viable blastocysts). Their results showed that media from viable blastocyst cultures contained significantly less methionine sulfoxide and lysophosphatidylcholine as compared to non-viable blastocyst media. Furthermore, viable blastocyst media also had lower concentrations of phospholipid, arginine, and methionine-related metabolites. The identified biomarkers can be further tested as to their ability to enhance embryo selection for transfer or cryopreservation.

Together, the studies presented in this Special Issue help extend our understanding of the regulation of early embryonic development at the molecular level and open new perspectives for the improvement and expansion of the current diagnostic methods related to fertility evaluation. Hopefully, they will also facilitate solutions to potential problems, especially in the context of in vitro fertilization. Special attention is paid to noninvasive approaches to reduce embryo damage and all its immediate consequences for embryos and long-term health risks for offspring.

